# Prognostic value of routinely assessed serum biomarkers in septic shock

**DOI:** 10.1186/cc9694

**Published:** 2011-03-11

**Authors:** E Lafuente, E Viegas, E Filipe, E Tomas, M Fernandes, J Gomes da Silva, F Santos, F Moura, R Lopes, P Santos, N Ribeiro, I Terra

**Affiliations:** 1 Centro Hospitalar Tamega e Sousa, Penafiel, Portugal

## Introduction

The objective of this study was to assess the usefulness of routinely admission measured biomarkers.

## Methods

From a sample of 256 patients enrolled between October 2009 and November 2010, 193 had sepsis and 63 had septic shock based on the ACCP/SCCM criteria, and for each of them we measured reactive protein C (RPC), total cholesterol, protein C activity (PC), albumin, arterial lactate and the levels of IL-6 at admission.

## Results

Levels of lactate, IL-6 and PC (< 40%) showed the best accuracy for prediction mortality in all of the study patients as much as in the arm of the septic shock patients (AUROC 0.76; 0.80; 0.75, respectively; and AUROC 0.86; 0.86; 0.75, respectively). See Table 1.

**Table 1:** 

	Septic shock	No septic shock	*P *value	No sepsis
SAPS II/mortality (%)	53.9 ± 19.1/52	35.1 ± 14.4/10	0.0001	30.2 ± 16/6.6
RPC	208 ± 115	185 ± 118	0.83	108 ± 105
Protein C	35.5 ± 17.8	56.1 ± 24	0.0001	65.8 ± 32.8
Albumin	2 ± 0.2	1.8 ± 0.6	0.5	2.3 ± 0.6
Lactate	4.5 ± 2.9	2.6 ± 2.6	0.0001	1.9 ± 1.8
Total cholesterol	77.8 ± 54	97.1 ± 48	0.02	136 ± 81
IL-6	42,252 ± 9,131	2,732 ± 725	0.001	1,434 ± 586

## Conclusions

Biomarkers at ICU admission revealed different accuracies in predicting septic shock mortality. Maximal lactate, mean IL-6 and minimum PC levels were associated with the higher mortality found in this ICU population.
